# DeepCE: a deep learning framework for correlation-enhanced gene regulatory network inference in single-cell RNA sequencing data

**DOI:** 10.1093/bioadv/vbag033

**Published:** 2026-01-30

**Authors:** Qianqian Wu, Xingmiao Dai, Shiyi Lou, Siyuan Wu, Tianhai Tian

**Affiliations:** School of Mathematics, Hefei University of Technology, Hefei, Anhui 230009, China; School of Mathematics, Hefei University of Technology, Hefei, Anhui 230009, China; School of Mathematics, Hefei University of Technology, Hefei, Anhui 230009, China; Computational BioMedicine Lab, College of Science and Engineering, James Cook University, Townsville, QLD 4188, Australia; School of Mathematics, Monash University, Melbourne, Wellington Road, Melbourne, VIC 3800, Australia

## Abstract

**Motivation:**

Single-cell RNA sequencing has substantially advanced our understanding of gene expression dynamics and cellular heterogeneity. In recent years, deep learning (DL) has emerged as a promising approach to infer genetic regulation. However, these methods still face challenges in representing complex regulatory mechanisms. Thus, it remains imperative to develop new algorithms to enhance both effectiveness and reliability.

**Results:**

We propose DeepCE, a DL framework for correlation-enhanced gene regulatory network (GRN) inference. DeepCE strengthens the extraction of dynamic regulation by integrating bidirectional gated recurrent units with convolutional neural networks (CNNs). Specifically, bidirectional gated recurrent units captures dynamic temporal dependencies, while CNNs focuses on local spatial patterns within single-cell data, enabling the model to uncover complex gene-gene interactions and generate high-quality GRNs. This framework improves the accuracy and robustness of GRN inference by smoothing noisy gene expression data, extracting time-lagged regulatory signals, and filtering out spurious correlations. Experiments conducted on mouse and human datasets demonstrate the strong performance of DeepCE. Performance evaluations show that DeepCE outperforms existing methods, achieving the highest AUROC and AUPR scores.

**Availability and implementation:**

Codes for DeepCE are free available in the GitHub https://github.com/sxiaodai/DeepCE.

## 1 Introduction

Recent advances in single-cell technologies have provided powerful tools for measuring molecular activities across thousands of individual cells simultaneously. A key advantage of single-cell studies is the ability to enable the detailed and accurate characterization of cellular heterogeneity (Farrell *et al.* 2018). However, the large volumes of high-dimensional and noisy single-cell data pose significant challenges to data analysis and mathematical modelling. The rise of single-cell data has catalyzed the computational and modelling approaches aimed at reconstructing dynamic cellular processes (Teschendorff and Feinberg 2021, Ding *et al.* 2022).

One of the central questions in single-cell analysis is to discover the mechanisms governing the dynamics of gene regulatory networks (GRNs) (Stumpf 2021). GRN inference methods are generally categorized into two classes: model-free approaches that rely on minimal assumptions, and mechanistic approaches based on dynamic system models (Nguyen *et al.* 2021). Most model-free methods utilize pairwise measures to infer undirected relationships between genes (Skinnider *et al.* 2019). These model-free techniques have also been extended to pseudo-time trajectory data, where causal inference methods are used to estimate directional regulatory interactions (Lu *et al.* 2021). However, relying solely on causality often yields suboptimal accuracy (Qiu *et al.* 2020). Enhancements such as incorporating RNA velocity (Liu *et al.* 2022) and accounting for time lags in gene expression (Specht and Li 2017) have improved performance. Nonetheless, simple similarity-based measures still struggle to capture the complex mechanisms underlying gene regulation.

Mechanistic approaches often employ mathematical/statistical models to represent the detailed dynamics of GRNs (Aubin-Frankowski and Vert 2020). Regularization techniques such as Lasso and Ridge regression have been widely used to infer sparse network structures (Omranian *et al.* 2016). In cases where time-series data are not available, single-cell data can still be utilized under the assumption that the timing of variables aligns with their predicted outcomes (Huynh-Thu *et al.* 2010). Evaluation frameworks such as BEELINE (Pratapa *et al.* 2020) have been developed to assess the accuracy of various GRN inference methods. Another comprehensive study benchmarked 13 inference techniques across 100 simulated datasets (Nguyen *et al.* 2021). More recently, ensemble approaches combining both model-free and mechanistic strategies have demonstrated improved robustness and performance (Ocone *et al.* 2015, Wang *et al.* 2016).

The integration of deep learning (DL) into biology has necessitated the development of adaptive data transformation frameworks that reconcile the complexity of biological systems with the requirements of neural network training. Specialized DL models have also been developed specifically for GRN inference. For instance, CNNC (Yuan and Bar-Joseph 2014) encodes gene expression data into matrices for convolutional feature extraction, while DeepFeature (Sharma *et al.* 2021) incorporates dimensionality reduction and class activation maps to optimize convolutional neural networks (CNNs) training. MTLRank (Song *et al.* 2023) integrates neural networks with multi-task learning to predict RNA velocity, thereby improving regulatory interaction accuracy. Similarly, DeepFGRN (Gao *et al.* 2024) combines bidirectional modules with correlation analysis to identify gene regulatory relationships and uncover potential biomarkers.

To further enhance GRN inference accuracy, we propose DeepCE, a DL framework specifically designed for correlation-enhanced GRN inference. The innovation is a sliding window mechanism to extract gene-gene associations, which incorporates transcription factor (TF) information and time-lag distributions to perform weak correlation filtering and strong correlation enhancement. The power of this process is to reduce noise and false associations effectively, providing more reliable inputs for the neural network. More importantly, DeepCE integrates Bidirectional Gated Recurrent Units (BiGRU) and CNN to capture both temporal and spatial features. This hybrid architecture enables the model to learn intricate patterns and accurately predict regulatory relationships, generating biologically meaningful GRNs.

## 2 Methods

**Figure 1:**
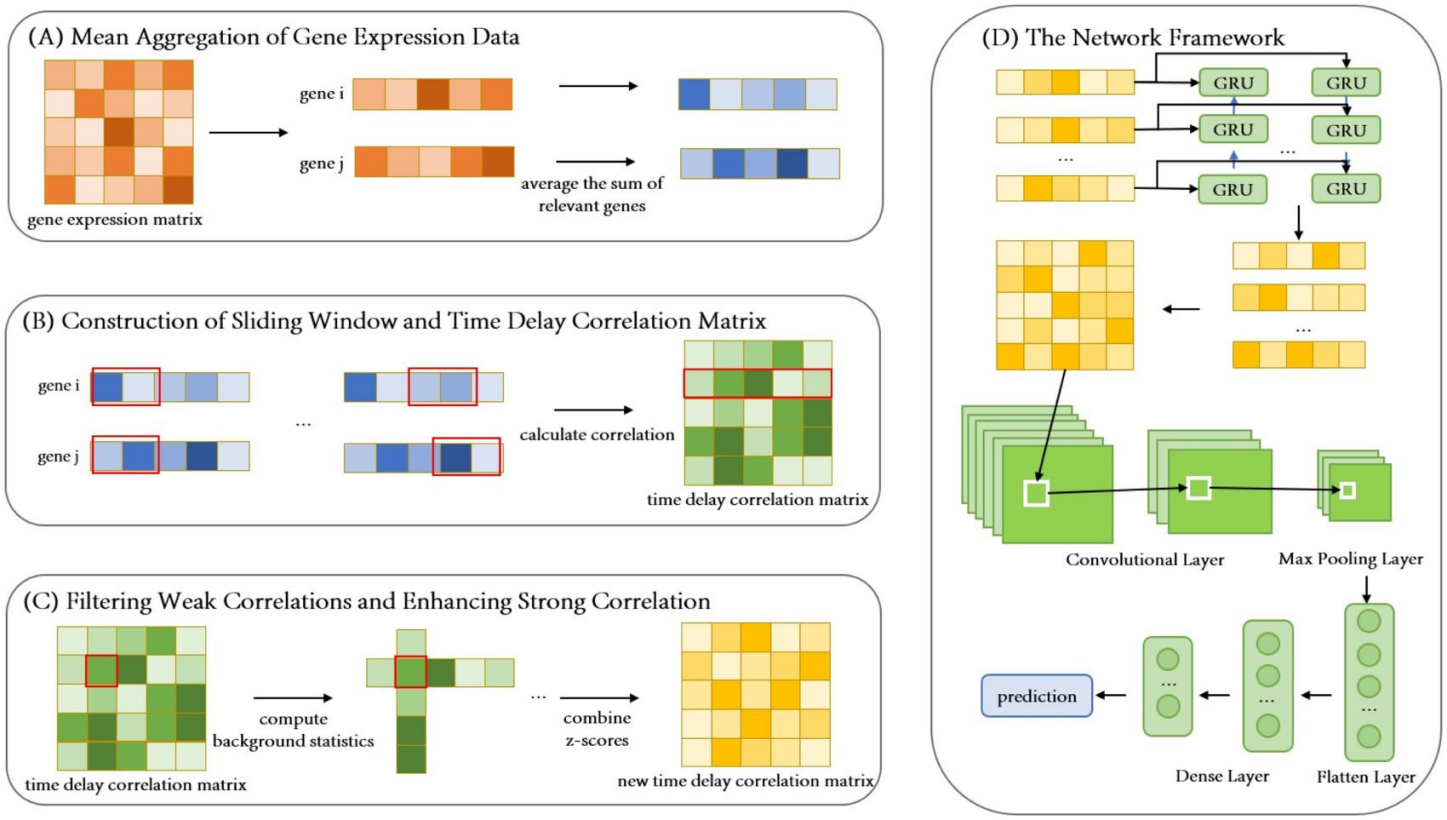
Framework of DeepCE. (A) Gene representations for TF *i* and target gene *j* are updated by averaging the expression vectors of their most strongly correlated genes. (B) Temporal correlations between gene segments of TF *i* and target gene *j*, incorporating time-lag relationships, are extracted to construct a time-delay correlation matrix. (C) Correlation values between gene segments are adjusted using two background distributions: one capturing the correlation between segments of TF *i* and target gene *j*, and the other reflecting correlations of gene fragments aligned at the same time delay. (D) The resulting refined correlation matrix is fed into a hybrid BiGRU–CNN model to extract temporal and spatial features for downstream regulatory prediction.

The proposed DL framework DeepCE is illustrated in Fig. 1. The framework comprises two main components: data preprocessing and model construction. This design enables the model to jointly capture temporal dynamics and spatial patterns from the refined correlation matrices, facilitating accurate prediction of regulatory interactions.

### 2.1 Dataset

This study utilizes three scRNA-seq datasets derived from independent experiments on mouse and human samples. The mouse hematopoietic stem and progenitor cells (mHSC) dataset (Nestorowa *et al.* 2016) comprises 1656 single-cell samples with expression profiles for 4773 genes. The human embryonic stem cell (hESC) dataset (Chu *et al.* 2016) includes 758 single-cell profiles spanning differentiation time points from 0 to 96 hours. The human hepatic cell (hHep) dataset (Camp *et al.* 2017) consists of 425 single-cell samples collected at five time points: 0, 6, 8, 14, and 21 days.

To evaluate the inference accuracy, we use experimentally validated or widely accepted ground truth networks for gene-gene and protein-protein interactions. We incorporate a variety of sources, including non-specific regulatory networks such as RegNetwork, TRRUST, and DoRothEA (Han *et al.* 2018, Garcia-Alonso *et al.* 2019), as well as cell-type-specific resources like ENCODE, ChIP-Atlas, and ESCAPE (Davis et al. 2018, Oki *et al.* 2018). Additionally, we used the STRING database for protein-protein interactions (Szklarczyk *et al.* 2019). Mouse and human datasets have been preprocessed using the BEELINE evaluation framework (Pratapa *et al.* 2020). Based on the variance ranking, the top 500 or 1000 genes are selected since they are typically closely associated with cell heterogeneity and key biological processes. Supplementary Information, available as supplementary data at *Bioinformatics Advances* online, provides more information for the selection of gene pairs and Table 1, available as supplementary data at *Bioinformatics Advances* online for summarizing the statistical information for the pre-processed datasets.

**Table 1:** Model performance across different network architectures.

Network architecture	mHSC-E (500)		mHSC-E (1000)	
	AUROC	AUPR	AUROC	AUPR
CNN	0.8374	0.4821	0.8221	0.3651
Simple RNN + CNN	0.8475	0.4941	0.8464	0.3719
LSTM + CNN	0.8454	0.4934	0.8466	0.3797
GRU + CNN	0.8465	0.4964	0.8537	0.3729
**BiGRU + CNN**	**0.8559**	**0.5281**	**0.8574**	**0.3891**
BiGRU	0.8232	0.4222	0.8081	0.3003
BiGRU + CNN64	0.8487	0.4937	0.8485	0.3782
**BiGRU + CNN64-32**	**0.8559**	**0.5281**	**0.8574**	**0.3891**
BiGRU + CNN64-32-64	0.8380	0.5200	0.8522	0.3795
BiGRU + CNN64-32-64-32	0.7598	0.2879	0.7734	0.2632

The bold values are the largest values derived from different methods.

### 2.2 Preprocessing pipeline

This study employs a preprocessing workflow centred on gene expression matrices and a curated gold-standard regulatory network. The primary aim is to identify high-value genes, highlight core regulatory interactions, and construct a balanced set of positive and negative gene-pair samples for downstream supervised learning.

Step 1: Loading and validating gene expression data. Raw gene expression data are first imported and subjected to basic quality-control checks to ensure completeness and suitability for subsequent regulatory network analysis.

Step 2: Removing low-expression genes (denoising). Genes expressed in ≤10% of cells (with the threshold defined as total_cells/10) are removed. This denoising step suppresses sparsely expressed genes and enhances the biological signal-to-noise ratio.

Step 3: Selecting highly variable genes. A variance-based ranking is applied to identify the top 1000 highly variable genes, which typically capture the majority of biologically relevant variation.

Step 4: Filtering the gold-standard regulatory network. For each TF-target gene pair in the gold-standard network, we retain only those interactions in which both the TF and the target gene appear in the selected top 1000 gene list. This focuses the analysis on core, high-confidence regulatory relationships.

Step 5: Constructing balanced positive and negative gene-pair samples. Positive samples correspond to validated TF–target pairs from the filtered gold-standard network. An equal number of negative samples (non-interacting TF–gene pairs) is generated to form a balanced dataset suitable for supervised machine learning task.

### 2.3 Mean aggregation of gene expression data

To mitigate the influence of random fluctuations and noise in gene expression data, we apply a gene expression aggregation method. Let the expression values of gene *i* after pseudotime ordering be $X_{i}=\left(x_{i,1},x_{i,2},\ldots ,x_{i,T}\right)$, where $x_{i,t}$ is the expression level of gene *i* in the *t*-th cell, and t∈[1,T]. Pearson correlation coefficient $\rho_{i,j}$ is used to quantify the similarity between the expression patterns of genes *i* and *j*. Then, we select *k* genes most highly correlated with gene *i* to highlight key regulatory signals. After selecting the top *k* genes, we then average their expression values as $\tilde{X_{i}}=\left({\tilde{x}}_{i,1},{\tilde{x}}_{i,2},\ldots ,{\tilde{x}}_{i,T}\right)$. This aggregated representation is used in downstream analysis to improve the stability and accuracy of GRN inference.

### 2.4 Construction of sliding window and time delay correlation matrix

To capture the delayed regulatory relationships between TFs and target genes, we adopt a sliding window strategy inspired by LEAP (Specht and Li 2017) and DGRNS (Zhao *et al.* 2022) (see Supplementary Information, available as supplementary data at *Bioinformatics Advances* online, for detail). For each TF–target gene pair, we apply the sliding window method to extract localized gene expression segments along the pseudotime axis. Let ${\tilde{X}}_{i,k}$ denote the expression segment of TF *i* in the *k*-th sliding window, spanning positions from (k−1)m+1 to (k−1)m+w. Similarly, ${\tilde{X}}_{j,l}$ represents the segment of target gene *j* aligned to the *l*-th window within the temporal context of TF *i*’s segment. This segment spans from (k−1)m+(l−1)n+1 to (k−1)m+(l−1)n+w.

For each pair of TF and target gene segments, we compute the Pearson correlation coefficient $\rho_{i_{k},j_{l}}$, which is stored in a matrix $M^{\left(i,j\right)}$ with element in *k*-th row and *l*-th column as


$$M_{k,l}^{\left(i,j\right)}=\rho_{i_{k},j_{l}},\;k=1,\ldots ,p;l=1,\ldots ,q.$$

Thus, each row of this matrix corresponds to a fixed segment of the TF, while each column represents a specific delay in the expression of the target gene. Parameters of the sliding window method are tuned to accommodate the characteristics of each dataset. For the mHSC-E dataset, we set p=q=64, m=n=5, and w=441. For the mHSC-L, mHSC-GM, hESC, and hHep datasets, the values of *w* are set to 217, 259, 128, and 173, respectively. Additionally, for the hHep dataset, the step sizes are adjusted to m=n=2 reflect its shorter trajectory.

### 2.5 Filtering weak correlations and enhancing strong correlations

Based on the Context Likelihood of Relatedness (CLR) method (Faith *et al.* 2007), we introduce modifications tailored to the characteristics of our time-delayed gene expression data (see Supplementary Information, available as supplementary data at *Bioinformatics Advances* online, for details). The CLR method uses a global background distribution of TF-target correlations. Here we propose two context-specific background distributions: one based on the correlations between a given TF fragment and all target gene fragments, and the other based on correlations of gene fragments aligned at the same time delay. This refinement allows the model to better capture regulatory trends of the same TF fragment across multiple target gene segments and to assess consistency across different time-delay conditions, ultimately improving signal stability and reducing noise. Note that our method adopts the conceptual idea of CLR background distributions but does not use actual CLR lookup tables.

For each correlation value $\rho_{i_{u},j_{u+v-1}}$ located at the *u*-th row and *v*-th column, we calculate the means and standard deviations (STD) of both the corresponding row and column. Let $\mu_{u}$ and $\sigma_{u}$ be the mean and STD of the *u*-th row. The standardized values are calculated as follows:


(1) $$z_{u,v}^{u}=\max\left(0,\frac{\rho_{i_{u},j_{u+v-1}}-\mu_{u}}{\sigma_{u}}\right),$$

(2) $$z_{u,v}^{v}=\max\left(0,\frac{\rho_{i_{u},j_{u+v-1}}-\mu_{v}}{\sigma_{v}}\right).$$

The integrated standardized value is obtained as follows:


(3) $$z_{i_{u},j_{v}}=\sqrt{{\left(z_{u,v}^{u}\right)}^{2}+{\left(z_{u,v}^{v}\right)}^{2}}.$$

Then we obtain a standardized matrix $Z^{\left(i,j\right)}={\left(z_{i_{u},j_{v}}\right)}_{p\times q}.$ The resulting matrix $Z^{\left(i,j\right)}$ is subsequently segmented and used as input for downstream neural network models to further learn and predict dynamic regulatory patterns.

### 2.6 Construction of deep learning model

In this study we use an integrated DL model that combines BiGRU and CNN modules to improve the inference of gene regulatory relationships. The BiGRU layer is configured with 128 neurons, and model hyperparameters are carefully tuned to minimize overfitting. Following the BiGRU, a CNN module is employed to extract local interaction patterns from the temporal features. The BiGRU output is reshaped into an appropriate format. Two convolutional layers, with 64 and 32 filters, respectively, are applied to learn hierarchical local features, followed by a max-pooling layer that reduces dimensionality. To prevent overfitting, dropout layers are incorporated. The resulting feature maps are then flattened and passed through three fully connected layers consisting of 512, 128, and 1 neuron, respectively, enabling high-order feature learning and producing the final prediction output.

The dataset is divided into training, validation, and test sets in a 3:1:1 ratio. We ensure the proportions of positive and negative samples in each subset to be consistent with those of the original dataset. The model is trained using binary cross-entropy as the loss function and optimized with stochastic gradient descent (SGD) with decaying learning rate decay. After training is complete, the final evaluation is conducted on the test set.

### 2.7 Assessment criteria

To assess model performance, we employ two standard metrics: the AUROC (area under the receiver operating characteristic curve) and AUPR (area under the precision-recall curve (AUPR). The ROC curve illustrates the relationship between the true positive rate (TPR) and false positive rate (FPR), which are defined in Supplementary Information, available as supplementary data at *Bioinformatics Advances* online.


(4) $$\text{TPR}=\frac{\text{TP}}{\text{TP}+\text{FN}},\;\text{FPR}=\frac{\;\text{FP}}{\text{FP}+\text{TN}},$$

where TP, TN, FP, and FN denote the numbers of true positives, true negatives, false positives, and false negatives, respectively.

In addition, AUPR measures the area under the Precision–Recall curve. Precision and recall are defined as


(5) $$\text{Precision}=\frac{\text{TP}}{\text{TP}+\text{FP}},\;\text{Recall}=\frac{\text{TP}}{\text{TP}+\text{FN}}.$$

Furthermore, we incorporate several early-retrieval and statistical-confidence metrics that better reflect the practical requirements of GRN inference. These include Precision@k, Early Precision, and false-discovery-rate (FDR) controlled subnetworks. Their definitions and mathematical formulations are provided in Supplementary Information. The results of these measures are also provided in Supplementary Information.

## 3 Results

### 3.1 Validation of gene regulatory temporal delays

To evaluate methods in capturing delayed regulatory effects, we used heatmap visualizations to assess whether the processed data accurately reflect true biological relationships. Six genes were selected for this analysis: MCM2 and MCM4 as TFs, and CDC7, ALG9, CDK1, and MYCN as target genes. Known regulatory interactions exist between MCM2–CDC7 and MCM4–CDK1, while no such relationships are reported for MCM2–ALG9 and MCM4–MYCN. Figure 1, available as supplementary data at *Bioinformatics Advances* online, presents time-delay correlation heatmaps for each gene pair after different stages of data processing.

When using the sliding window method alone, the heatmaps for the regulated pairs MCM2–CDC7 and MCM4–CDK1 exhibit high-correlation regions in the upper-left and lower-right corners. In contrast, the non-regulated pairs MCM2–ALG9 and MCM4–MYCN dis-play different patterns. While MCM2–ALG9 shows a few isolated high-value regions, MCM4–MYCN exhibits no significant correlations, which is consistent with the absence of regulatory interaction.

When all processing methods are combined, the heatmaps for the regulated gene pairs show even more clearly defined distinctions between high- and low-value regions. For the non-regulated pair MCM2–ALG9, high-value patches become more fragmented after weak correlation filtering, while for MCM4–MYCN, the elimination of high-value regions suggests absent regulatory associations. We also test the processing methods together with mean aggregation or with weak correlation filtering and strong correlation enhancement, results are presented in Supplementary Information, available as supplementary data at *Bioinformatics Advances* online. More detailed analysis for these four types of processing methods is provided in Supplementary Information, available as supplementary data at *Bioinformatics Advances* online. Overall, the combined data processing pipeline improves the resolution of regulatory signal patterns between gene segments and enables more effective differentiation between gene pairs with and without regulatory relationships.

### 3.2 Impact of network architectures on performance

To evaluate the contributions of the BiGRU and CNN modules to model performance, we conducted ablation experiments on two datasets: mHSC-E (1000) and mHSC-E (500). Each configuration was tested over 10 independent runs using fixed dataset splits to ensure consistency and minimize variability. The goal was to examine the individual and combined effects of BiGRU and CNN on prediction accuracy, as measured by AUROC and AUPR. The results are summarized in Table 1.

We first assessed the effect of the BiGRU component by comparing the BiGRU + CNN model with alternative RNN-CNN architectures, including Simple RNN + CNN, LSTM + CNN, GRU + CNN, and a CNN-only model (upper half of Table 1). Across both datasets, BiGRU + CNN consistently outperformed all other configurations in terms of both AUROC and AUPR. Specifically, compared to the CNN-only baseline, BiGRU + CNN achieved a 2.69% improvement in AUROC and a 3.50% increase in AUPR. While the improvement over other RNN-based architectures were more modest in AUROC (∼0.90%), the gain in AUPR was more notable, with a 2.39% increase.

The CNN-only model tended to overfit, likely due to its limited ability to capture long-range dependencies in sequential data. In contrast, RNN-based architectures—particularly BiGRU—better modeled temporal dynamics and reduced overfitting. The superior performance of BiGRU is attributed to its bidirectional structure, which captures both past and future context, and its efficient gating mechanism that improves long-term dependency modeling. Based on these results, BiGRU was selected as the recurrent backbone of the final model.

We next evaluated the impact of the CNN module, which consisted of two convolutional layers with 64 and 32 filters, followed by max pooling. This architecture is denoted as BiGRU + CNN64-32 (lower half of Table 1). We compared this configuration against several variants, including BiGRU alone, BiGRU + CNN64, BiGRU + CNN64-32–64, and BiGRU + CNN64-32–64-32, to investigate the influence of CNN depth on performance.

BiGRU + CNN64-32 achieved the best overall performance, improving AUROC by 4.10% and AUPR by 9.74% compared to BiGRU alone. Compared to BiGRU + CNN64 and BiGRU + CNN64-32–64, the improvements were more modest—0.98% in AUROC and 1.58% in AUPR—indicating that the two-layer CNN strikes an effective balance between complexity and representational power. Notably, the deeper architecture BiGRU + CNN64-32-64-32 performed the worst, even underperforming BiGRU alone. This suggests that excessive CNN depth may introduce redundancy or lead to overfitting and information loss.

These results demonstrate that a moderate CNN depth improves the model’s ability to extract local features and enhances predictive accuracy. BiGRU + CNN64-32 provides an optimal trade-off between model complexity, training efficiency, and performance, and is therefore adopted as the final architecture.

### 3.3 Impact of data preprocessing on performance

We first investigate the impact of the aggregation parameter *k* on the model performance. We tested different values of *k* on the mouse datasets, with results summarized in Table 2. For mHSC-E (500), the optimal performance was achieved at k=10. In mHSC-L (500), increasing *k* led to a decline in AUROC but an increase in AUPR, suggesting improved detection of positive samples but diminished ability to distinguish negatives. For mHSC-GM (500), the best performance occurred at k=5. In the 1000-gene datasets, mHSC-E (1000) showed increasing AUROC with larger *k*, although AUPR decreased, implying reduced precision in identifying true positives. For mHSC-L (1000), k=10 and k=5 provided a good trade-off between information enrichment and redundancy. In mHSC-GM (1000), the highest performance was observed at k=10. These findings demonstrate that the optimal aggregation number *k* varies across datasets. Therefore, careful tuning is essential to balance the trade-off between preserving informative patterns and avoiding redundancy, ultimately enhancing model robustness and prediction accuracy.

**Table 2:** Model performance under different mean aggregation numbers *k*.

*k*	mHSC-E (500)		mHSC-L (500)		mHSC-GM (500)		mHSC-E (1000)		mHSC-L (1000)		mHSC-GM (1000)	
	AUROC	AUPR	AUROC	AUPR	AUROC	AUPR	AUROC	AUPR	AUROC	AUPR	AUROC	AUPR
5	0.6875	0.1640	**0.7245**	0.1568	**0.7403**	**0.3618**	0.7374	0.1370	0.6425	0.1059	0.7097	0.1594
10	**0.7142**	**0.2235**	0.7181	0.1363	0.6903	0.3036	0.7583	**0.1423**	**0.6469**	0.1198	**0.7579**	**0.2044**
15	0.6540	0.1535	0.6706	**0.2227**	0.6691	0.1401	0.7687	0.1333	0.6433	**0.1226**	0.7053	0.1098
20	0.6000	0.1402	0.6369	0.2083	0.5372	0.1459	**0.7777**	0.1304	0.6326	0.1197	0.6596	0.0671

The bold values are the largest values derived from different methods.

To evaluate the effectiveness of the proposed data preprocessing methods, we conducted experiments on the mouse datasets under four different configurations: (i) sliding window only, (ii) mean aggregation combined with sliding window, (iii) sliding window with weak correlation filtering and strong correlation enhancement, and (iv) a combination of all three methods. The results are summarized in Table 3. Among the four configurations, the inclusion of either mean aggregation or correlation-based enhancement led to clear improvements in both AUROC and AUPR, demonstrating that each method contributes uniquely to enhancing the quality of extracted features. Notably, the combined approach—which integrates all three preprocessing techniques—achieved the best overall performance.

**Table 3:** Model performance under different data preprocessing methods (baseline: using sliding window only; aggregation: using mean aggregation with sliding window; adjustment: using sliding window with weak correlation filtering and strong correlation enhancement; combined: all methods combined).

Method	mHSC-E (500)		mHSC-L (500)		mHSC-GM (500)		mHSC-E (1000)		mHSC-L (1000)		mHSC-GM (1000)	
	AUROC	AUPR	AUROC	AUPR	AUROC	AUPR	AUROC	AUPR	AUROC	AUPR	AUROC	AUPR
Baseline	0.6346	0.1705	0.5265	0.1330	0.6052	0.1547	0.6001	0.1323	0.6403	0.1133	0.6540	0.0992
Aggregation	0.7142	0.2235	0.7181	0.1363	0.6903	0.3036	0.7583	0.1423	0.6469	0.1198	0.7579	0.2044
Adjustment	0.7190	0.2160	0.6896	0.1560	0.6809	0.1841	0.7505	0.2257	0.6653	0.1308	0.6904	0.1708
Combined	**0.8559**	**0.5281**	**0.8140**	**0.3358**	**0.7751**	**0.3976**	**0.8574**	**0.3891**	**0.7734**	**0.2656**	**0.8291**	**0.2581**

The bold values are the largest values derived from different methods.

On the mHSC (500) datasets, the combined method improved AUROC and AUPR by an average of 22.62% and 26.78%, respectively, compared to using the sliding window alone. When compared to the individual use of either mean aggregation or correlation-based enhancement, the combined method yielded average gains of 11.30% in AUROC and 21.73% in AUPR. Similar trends were observed on the mHSC (1000) dataset, where the combined method increased AUROC and AUPR by 18.85% and 18.93% over the sliding window baseline, respectively. Compared to the individual enhancement method, the combined method increased AUROC and AUPR by 10.84% and 13.86%, respectively. These results confirm that integrating multiple preprocessing strategies significantly improves the model’s ability to extract meaningful regulatory signals and enhances overall prediction performance.

To assess the influence of potential trajectory bias arising from pseudotime inference, we performed a perturbation analysis by randomly modifying the pseudotime of each cell according to


$$t_{i}^{\prime }=t_{i}+\mu N(0,1).$$

where μ controls the perturbation magnitude. For the mHSC-E dataset containing 1071 cells, the average numbers of positional shifts along the trajectory were 148 and 219 when μ=0.1 and μ=0.2, respectively. The corresponding performance values (AUROC, AUPR) were (0.818, 0.396) for μ=0.1 and (0.806, 0.369) for μ=0.2. Compared with the unperturbed performance of (0.856, 0.528), these results indicate that DeepCE remains reasonably robust under substantial perturbations of the pseudotime trajectory.

To assess the influence of the similarity measure on inference accuracy, we employed mutual information, Spearman and Kendall correlation coefficients to select the top k=10 genes for mean aggregation of expression data using the mHSC-E dataset. The results (Table 4, available as supplementary data at *Bioinformatics Advances* online) demonstrate close agreement with those obtained using Pearson’s *r* in Table 3. This agreement arises because the majority of the top *k* genes identified by the two measures are identical. Moreover, the remaining differing genes still exhibit considerable similarity in their expression profiles. Consequently, when the mean expression values of these top *k* genes are used to represent the expression of the gene of interest, the aggregated values derived from different similarity measures are nearly indistinguishable.

### 3.4 Comparison of different inference methods

**Figure 2:**
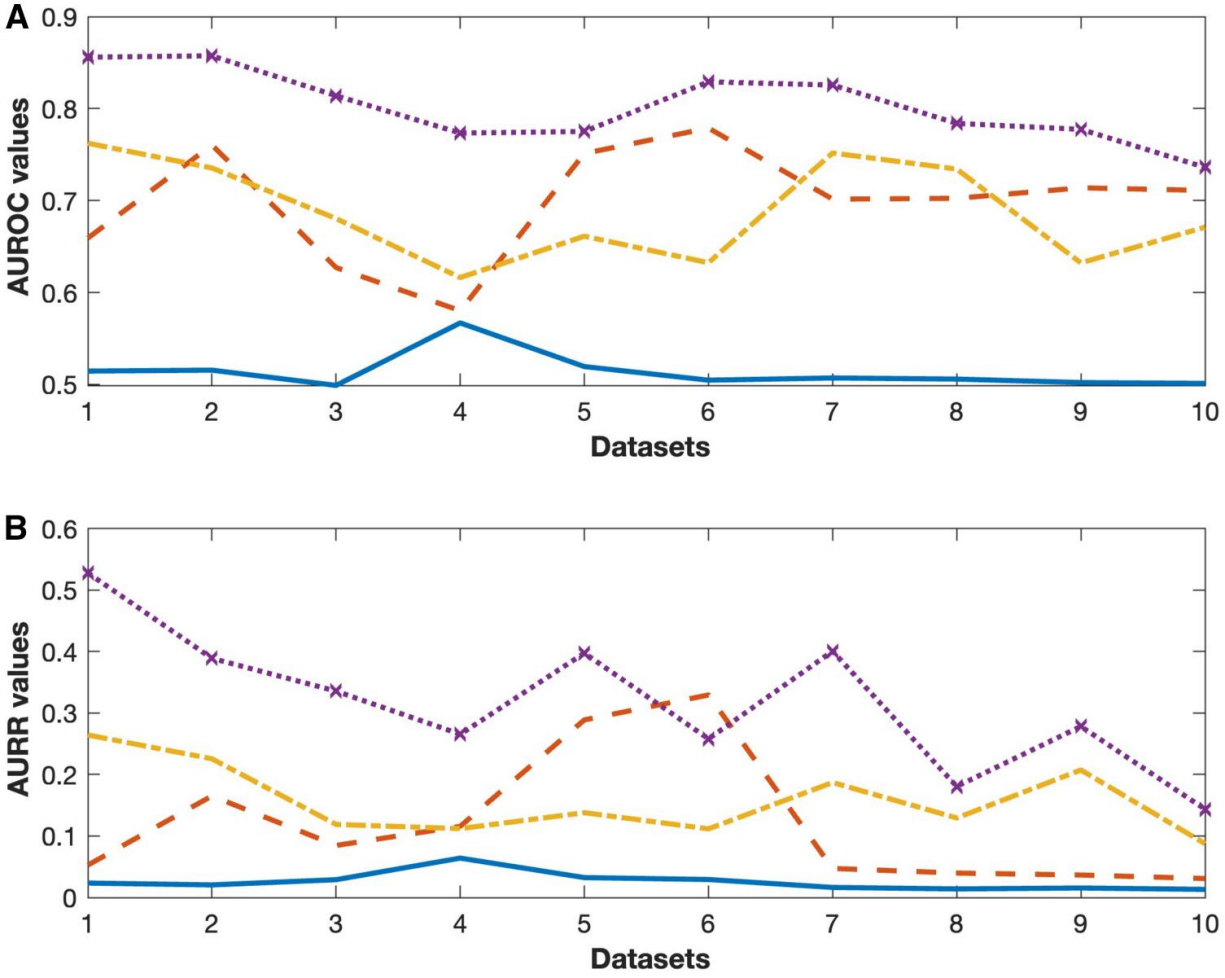
Comparison of model performance across different methods on the mouse and human datasets. (A) AUROC values. (B) AUPR values. (Dataset indexes: 1. mHSC-E (500); 2. mHSC-E (1000); 3. mHSC-L (500); 4. mHSC-L (1000); 5. mHSC-GM (500); 6. mHSC-GM (1000); 7. hHep (500); 8. hHep (1000); 9. hESC (500); 10. hESC (1000)). (Solid-line: DeepSEM, dash-line: GENELink, dash-dot line: DGRNS, dot-line: DeepCE).

To validate the effectiveness of our proposed method, we compared it against three state-of-art DL-based inference approaches, namely DeepSEM (Shu *et al.* 2021), GENELink (Chen and Liu 2022), and DGRNS (Zhao *et al.* 2022). The performance of these methods on mouse and human datasets is presented in Fig. 2. It shows that DeepCE outperforms the other methods on most mouse datasets. On average, DeepCE achieves an AUROC improvement of 29.75% and an AUPR improvement of 32.93% over DeepSEM, a 12.47% higher AUROC and 18.96% higher AUPR than GENELink, and a 13.62% increase in AUROC and 20.06% increase in AUPR compared to DGRNS.

DeepSEM shows the weakest performance overall, primarily due to its unsupervised nature and lack of reliance on biological priors. While advantageous in data-scarce settings, its inference accuracy suffers from the absence of regulatory context. GENELink, which incorporates prior regulatory knowledge, performs well when prior information is accurate and data are complete, but is vulnerable to performance degradation in the presence of noisy or incorrect priors. DGRNS, which uses pseudo-time ordering and sliding windows to capture spatiotemporal dependencies, is also sensitive to noise and data sparsity, which can hinder its ability to extract reliable regulatory patterns.

For human datasets, DeepCE continues to outperform the three baseline methods. Specifically, compared to DeepSEM, DeepCE achieves an average improvement of 27.68% in AUROC and 23.61% in AUPR. Relative to GENELink, AUROC and AUPR increase by 7.36% and 21.23%, respectively. When compared to DGRNS, DeepCE delivers improvements of 8.35% in AUROC and 9.80% in AUPR. The consistently strong results of DeepCE on human datasets not only validate the model’s effectiveness and robustness but also underscore its potential for cross-species generalization, making it a promising tool for broader applications in biomedical research.

We have also incorporated additional evaluation metrics—Precision@k, Early Precision, and FDR-controlled subnetwork analysis—into the Supplementary Information, available as supplementary data at *Bioinformatics Advances* online. The corresponding results are reported in Tables 5 and 6, available as supplementary data at *Bioinformatics Advances* online. Table 5, available as supplementary data at *Bioinformatics Advances* online, presents the performance of our proposed method DeepCE across three mouse datasets, demonstrating that the method yields consistently robust results across different experimental conditions. Table 6, available as supplementary data at *Bioinformatics Advances* online, further compares the performance of various methods on the mHSE-E(500) dataset with *k* = 10. Our method DeepCE ranks immediately after GeneLink, which achieves the best overall performance across the three-evaluation metrics.

### 3.5 Prediction results presentation

**Figure 3:**
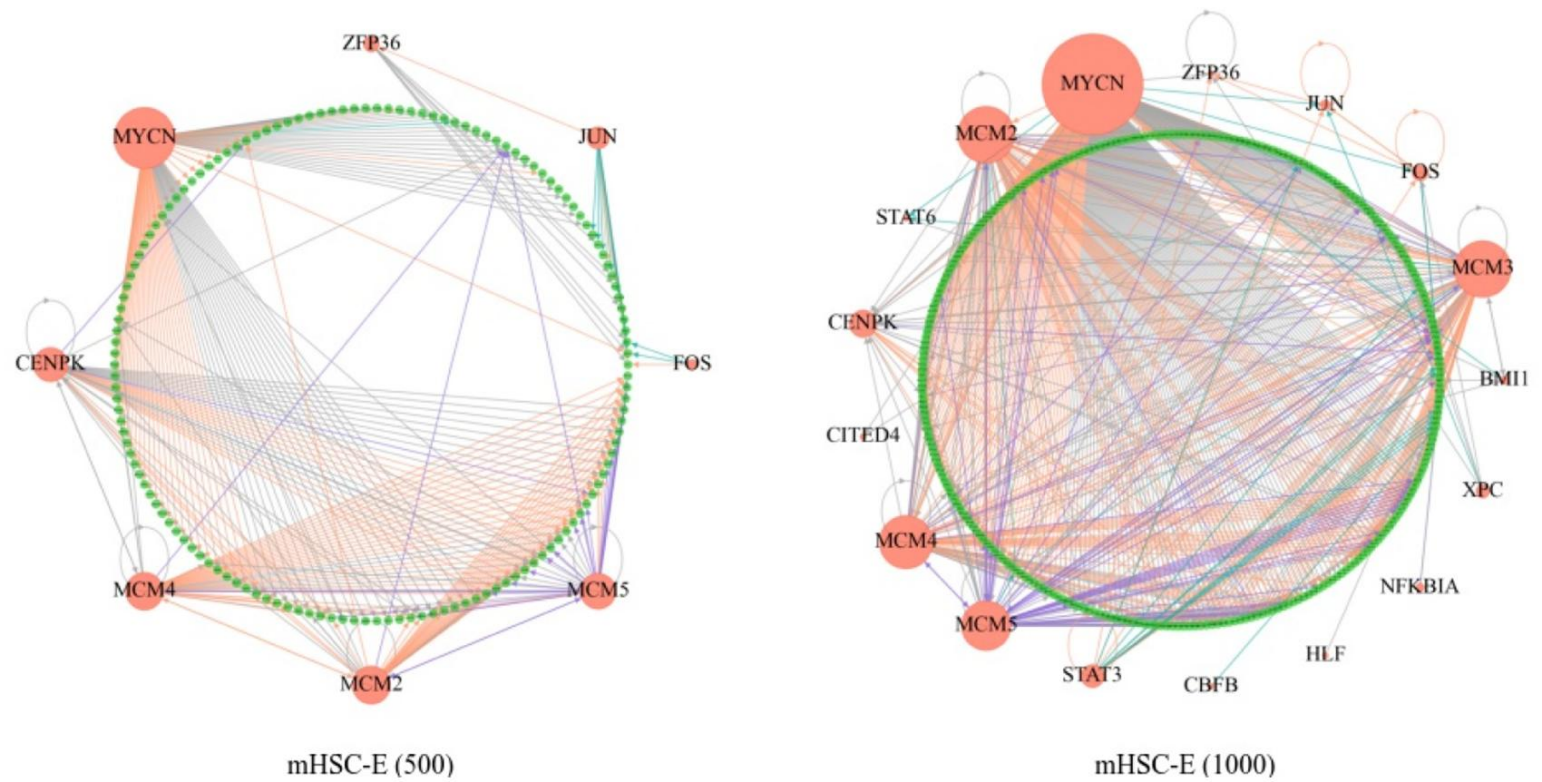
Diagrams of predicted gene regulatory networks (mHSC-E datasets). Left: GRN for mHSC-E (500). Right: GRN for mHSC-E (1000).

Finally, we performed predictions using the trained model on each respective dataset. Based on the predicted probabilities, we ranked all gene pairs in descending order and selected the top-ranking pairs to match the number of known regulatory interactions in each dataset. For the mHSC-E datasets, Fig. 3 presents the predicted GRNs for both mHSC-E (500) and mHSC-E (1000). Directed edges between nodes denote inferred regulatory relationships. The number of key TFs identified in mHSC-E (500) is slightly lower than in mHSC-E (1000), likely due to the reduced gene set. However, there is substantial overlap between the two networks. TFs such as MYCN, MCM2, MCM4, and JUN consistently appear across both datasets and are frequently involved in regulatory interactions, highlighting their prominent roles in hematopoietic stem cell regulation. The size of each TF node is scaled proportionally to the number of regulatory connections it possesses. Predicted GRNs for the remaining datasets are provided in Supplementary Information, available as supplementary data at *Bioinformatics Advances* online.

The results demonstrate that the model successfully identified known regulatory interactions documented in the non-specific network, which are highlighted with red edges. In addition, our model predicted regulatory relationships not present in the non-specific network. Among these, blue edges indicate interactions supported by cell-type-specific networks, purple edges correspond to those validated in the STRING database, and grey edges represent interactions not recorded in any of the three reference networks. These may reflect data limitations, model-specific inference artifacts, or previously uncharacterized biological relationships.

To investigate the biological underpinnings of the genetic regulation patterns identified by DeepCE, we conducted functional enrichment analysis on the top regulatory pairs (Fig. 4, available as supplementary data at *Bioinformatics Advances* online). Inte-grated GO and KEGG analysis of the top 50 high-loading genes in Fig. 4a, available as supplementary data at *Bioinformatics Advances* online, reveals a multifaceted and highly interconnected functional module. KEGG pathway analysis shows significant enrichment in pathways related to the cell cycle, DNA replication, pyrimidine metabolism, and nucleotide metabolism—core modules closely associated with cellular proliferation and genome maintenance. In addition, Fig. 4b, available as supplementary data at *Bioinformatics Advances* online, visualizes the enrichment results by integrating statistical significance with the corresponding enrichment magnitude. The integrated Gene Ontology analysis in Fig. 4c, available as supplementary data at *Bioinformatics Advances* online, highlights multiple coherent modules across biological processes, cellular components, and molecular functions. Finally, Fig. 4d, available as supplementary data at *Bioinformatics Advances* online, illustrates the gene–pathway relationships, depicting how individual genes map onto the top enriched biological pathways.

## 4 Conclusion

In this work, we propose DeepCE, a novel framework for improving the inference accuracy of GRN. To address the issue of data sparsity, we introduce a mean aggregation method that averages the expression profiles of the most highly correlated genes, effectively approximating underlying expression trends. To capture time-delayed regulatory effects, we construct a time-delay correlation matrix using a sliding window approach. This enables the extraction of directionality and similarity in regulatory trends across different time points. We further refine the matrix by filtering out weak associations and enhancing strong correlations, reducing the influence of spurious relationships on network inference.

Our model architecture combines BiGRU with CNN to jointly capture temporal dependencies and local expression patterns. The BiGRU component models dynamic regulatory dependencies across varying time lags, making it well-suited for sequential gene expression data. The CNN component complements this by extracting spatially local expression features, thereby enriching the model’s ability to capture complex regulatory signals. This hybrid architecture significantly improves performance over existing models and inference methods, yielding more accurate and biologically meaningful GRNs, including the potential identification of novel regulatory relationships.

The core methodology assumes that gene regulatory relationships manifest as time-delayed correlations along cellular trajectories. This trajectory-dependence creates two key limitations: First, we cannot employ standard conditional independence tests (e.g. partial correlation given pseudotime) because removing trajectory structure invalidates the temporal framework essential for measuring delays. Second, our method is inherently sensitive to trajectory inference quality and may identify spurious time-lagged relationships between genes that are independently activated at different phases of the same process. To address these two limitations, future work should develop trajectory-aware methods. For the first issue, advanced null models that preserve gene autocorrelation while disrupting phase relationships are needed. For the second, integrating chromatin accessibility and motif data will help distinguish direct regulation from co-activation. Multi-trajectory consensus approaches could further separate robust signals from trajectory artifacts.

Future work may also involve developing more robust denoising techniques and incorporating correlation measures to better capture nonlinear dependencies. In addition, exploring alternative network architectures and integrating multi-omics data could further enhance our understanding of regulatory mechanisms, particularly in the context of disease research and precision medicine.

## Supplementary Material

vbag033_Supplementary_Data

## Data Availability

Codes for DeepCE are free available in the GitHub https://github.com/sxiaodai/DeepCE
