# Supplementary material for: DeepCE: a deep learning framework for correlation-enhanced gene regulatory network inference in single-cell RNA sequencing data
Source: Bioinform Adv. 2026 Jan 30;6(1):vbag033. doi: 10.1093/bioadv/vbag033 (PMC12916171; doi:10.1093/bioadv/vbag033)
Supplement: vbag033_Supplementary_Data [file vbag033_supplementary_data.pdf]

Supplementary Information
DeepCE: a deep learning framework for correlation-enhanced gene regulatory network inference in single-cell RNA sequencing data

December 22, 2025

1 Statistical summary of experimental data

This study utilized three scRNA-seq datasets derived from experiments on both mice and humans. The mouse hematopoietic stem and progenitor cell (mHSC) dataset, reported by Nestorowa et al.[7], comprises scRNA-seq profiles of 1,656 mHSCs with expression measurements for 4,773 genes. The human embryonic stem cell (hESC) dataset, provided by Chu et al.[2], consists of 758 scRNA-seq samples spanning differentiation time points from 0 to 96 hours. The human hepatic cell (hHep) dataset, from Camp et al. [1], includes 425 scRNA-seq samples collected at 0, 6, 8, 14, and 21 days.

Statistical summaries of cell counts, gene counts, and gene expression values for each dataset are presented in Table 1. Notably, the reported zero rates underscore the pronounced sparsity and noise inherent to single-cell data, key challenges that must be addressed in downstream analyses.

STable 1. Statistical Information of Mouse and Human Single-Cell Gene Expression Data

Dataset	Cells	Genes	Mean	Std Dev	Min	Max	Zero Rate
mHSC-E	1071	4762	2.4866	2.1851	0	18.6162	0.4254
mHSC-L	847	4762	2.3815	2.1684	0	15.9751	0.4373
mHSC-GM	889	4762	2.4467	2.1585	0	17.2984	0.4240
hESC	758	17735	2.3357	1.5576	0	19.3457	0.5098
hHep	425	11515	2.2361	1.8063	0	18.3948	0.4685

Ground truth networks serve as benchmarks for evaluating prediction methods and models, providing a reference to validate the accuracy of inferred results. In this study, we employed multiple network data sources, including non-specific networks, cell-specific networks, and STRING networks. Non-specific networks encompass general TF–target gene regulatory

relationships applicable across diverse biological systems. These networks are primarily derived from databases such as RegNetwork [6], TRRUST [5], and DoRothEA [4], which include experimentally validated and computationally predicted TF–target interactions. Cell-specific networks, obtained from resources such as ENCODE [3] and ChIP-Atlas [8], emphasize TF regulation within specific cell types, enabling the analysis of regulatory heterogeneity between different cellular contexts. STRING networks, sourced from the STRING database [11], focus on protein–protein interactions (PPIs) and integrate data from experimental results, computational predictions, and literature mining.

Table 2 summarizes the key characteristics of these networks. In our framework, non-specific networks were used for model training, as they capture broad gene regulatory relationships applicable across multiple cell types. To evaluate prediction reliability, we further compared our results against cell-specific and STRING networks.

STable 2. Statistical Information of the Ground Truth Networks

Network	Genes	TFs	Target Genes	Regulations	Reference
Mouse Non-Specific Network	17,854	1,456	17,831	100,140	[5, 6]
mHSC Cell-Specific Network	19,328	138	19,324	1,078,889	[3, 8]
Mouse STRING Network	7,773	1,351	7,637	157,135	[11]
Human Non-Specific Network	23,568	2,166	23,381	386,294	[4–6]
hESC Cell-Specific Network	18,106	131	18,105	436,564	[3, 8, 12]
hHep Cell-Specific Network	16,824	85	16,823	342,863	[3, 8]
Human STRING Network	8,808	1,490	8,713	198,286	[11]

To reconstruct the temporal sequence of cells in biological processes, we first arrange the cells based on pseudotime to reflect their dynamic changes. The data preprocessing follows the BEELIN evaluation framework proposed by Pratapa et al. [9]. Specifically, after obtaining the pseudotime values, a generalized additive model (GAM) is used to compute the variance of gene expression over pseudotime and assess its significance (p), and then the Bonferroni correction is applied to control for multiple hypothesis testing. Based on the variance ranking, we select the top 500 or 1000 genes, as these are typically closely associated with cell heterogeneity and key biological processes. To minimize the impact of lowly expressed genes on the analysis, the genes selected should exclude those that are expressed in fewer than 10% of the cells and those with $p \geq 0.01$. Then only genes in gene pairs which appear in the ground truth network will be considered and recorded. Finally, we log-transform and normalize the data to reduce the impact of sequencing depth differences and ensure data stability and consistency. After this processing, the selected gene set not only reduces noise but also enhances biological interpretability, providing a more reliable input for subsequent GRN inference. Table 3 summarizes the statistical information of the preprocessed datasets.

STable 3. Statistical summary of preprocessed mouse and human single-cell gene expression datasets

Dataset	Genes	Cells	TFs	Regulations	Avg. TF-Targets	Network Density
mHSC-E (500)	169	1071	21	279	13.2857	0.0786
mHSC-E (1000)	354	1071	45	676	15.0222	0.0424
mHSC-L (500)	114	847	22	147	6.6818	0.0586
mHSC-L (1000)	403	847	63	845	13.4127	0.0333
mHSC-GM (500)	108	889	17	154	9.0588	0.0839
mHSC-GM (1000)	321	889	48	566	11.7917	0.0367
hESC (500)	188	758	22	236	10.7273	0.0571
hESC (1000)	541	758	55	808	14.6909	0.0272
hHep (500)	90	425	17	105	6.1765	0.0686
hHep (1000)	258	425	40	346	8.6500	0.0335

2 Sliding Window Method

In DGRNS [13], a sliding window method derived from LEAP [10] is utilized to capture time-lagged information in gene regulation. Unlike the LEAP, which employs a fixed window for TFs, DGRNS addresses this limitation by simultaneously applying sliding windows to both TFs and target genes. This enhancement significantly improves the accuracy and flexibility in modeling time-delayed regulatory relationships.

The method divides the pseudotime axis into local windows. For TF i, the window starts at the beginning of the pseudotime axis, covering w cells and sliding right with a step size of m. For target gene j, the window begins aligned with the TF window but slides with a step size of n. The resulting gene segments for the gene pair can be expressed as:

$$X_{i,a} = \left(x_{i,(a-1)m+1}, \ldots, x_{i,(a-1)m+w}\right), \tag{2.1}$$

$$X_{j,b} = \left(x_{i,(a-1)m+(b-1)n+1}, \ldots, x_{i,(a-1)m+(b-1)n+w}\right). \tag{2.2}$$

Here, $X_{i,a}$ and $X_{j,b}$ denote the gene segments captured by the a-th and b-th sliding windows of TF i and target gene j, respectively. Then calculate the Pearson correlation coefficient $\rho\left(X_{i,a}, X_{j,b}\right)$ between each pair of TF and target gene segments. This method quantifies the correlation between gene segment pairs, which is essential for capturing the time-lagged relationships in gene regulation.

Define q as the number of sliding windows for the TF, and p as the number of sliding windows for the target gene, where each target gene sliding window corresponds to a TF sliding window with a different time delay. To ensure that the last sliding window of the TF still corresponds to a sufficient number of target gene sliding windows, the following constraint must be satisfied:

$$T = w + q(m-1) + p(n-1). \tag{2.3}$$

where T is the total number of cells ordered by pseudotime. This formulation ensures that each TF window can be meaningfully paired with target gene segments across multiple time delays, enabling more accurate inference of regulatory direction and temporal dynamics.

3 Validation of Gene Regulatory Temporal Delays

We use the sliding window method to verify the effectiveness of the data processing methods used for extracting gene regulation lag effects. In addition, we employed methods like mean aggregation, weak correlation filtering and correlation enhancement to address issues of data sparsity and noise. Mean aggregation helps alleviate sparsity by averaging the expression values of multiple related genes, but this method may introduce additional correlation information, potentially affecting the accuracy of the data. Meanwhile, in weak correlation filtering and correlation enhancement, there is still uncertainty about whether the chosen distributions effectively capture true correlations.

To evaluate the effectiveness of various data processing methods in capturing the lag effects of gene regulation, we assessed the processed data using heatmaps to determine whether they accurately reflect true regulatory relationships. For gene pairs with known regulatory interactions, such as MCM2–CDC7 and MCM4–CDK1, Fig.1 presents time-lag correlation matrix heatmaps generated using different processing approaches. In each subplot, the vertical axis represents TF segments (top to bottom), and the horizontal axis represents target gene segments (left to right). Figures1(a) and 1(b) show the heatmaps of MCM2–CDC7 and MCM4–CDK1 processed solely with the sliding window method. Figures 1(c) and 1(d) display results obtained after applying mean aggregation followed by the sliding window method. Figures 1(e) and 1(f) depict heatmaps processed using a combination of sliding window, weak correlation filtering, and correlation enhancement. Finally, Figures 1(g) and 1(h) show the results of applying all techniques together—mean aggregation, sliding window, weak correlation filtering, and correlation enhancement.

Across the two gene pairs, the heatmaps reveal similar trends. When using only the sliding window method, the upper-left and lower-right corners of Figures 1(a) and 1(b) display prominent high-value regions, indicating that early- and mid-stage TF expression influences the corresponding stages of target gene expression, while late-stage TF fluctuations primarily affect target gene expression in later stages. These patterns demonstrate that TF–target gene expression is not fully synchronous, confirming the time-lagged nature of regulatory interactions. The high-value regions occupy only specific parts of the plots, suggesting that TF regulation occurs during discrete periods. Additionally, the elevated values on the far left indicate a strong alignment between TF and target gene segments, implying that regulatory gene pairs exhibit similar dynamic patterns, which supports the rationale for mean aggregation.

After applying mean aggregation (Figures 1(c) and 1(d)), the high-value regions persist, showing that this method effectively preserves time-lag information. The left-aligned correlation, however, becomes more pronounced, likely due to the replacement of single-gene expression values with aggregated signals from multiple related genes, which amplifies correlation, espe-

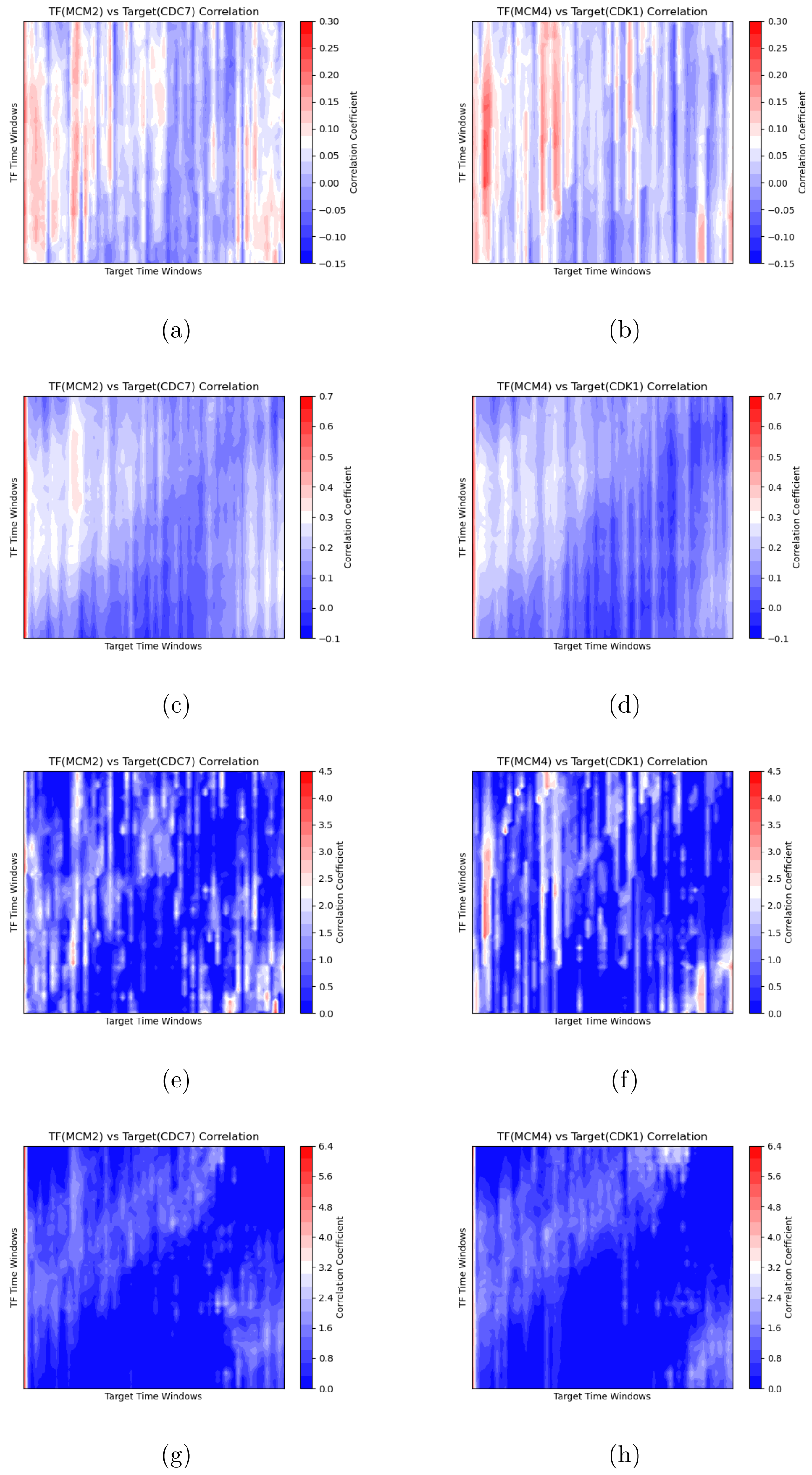

SFigure 1. Time-lagged correlation matrix heatmap for regulated gene pairs

cially at initial positions. When gene expression trends are similar, this aggregation enhances the prominence of correlated regions. Compared to Figures 1(a) and 1(b), mean aggregation reduces noise, smooths correlation profiles, and highlights key high-value regions, confirming its utility. Although the elevated correlation on the left may influence subsequent analyses, its effect is limited because it is confined to the first sliding window, and neural networks primarily focus on learning global rather than local features.

Following weak correlation filtering and correlation enhancement (Figures 1(e) and 1(f)), the contrast between high- and low-value regions becomes sharper, though at the cost of reduced continuity. Nevertheless, the persistence and clarity of high-value regions confirm the method's effectiveness. Finally, combining all methods (Figures 1(g) and 1(h)) yields the advantages of each: greater contrast between high- and low-value regions, further amplified correlations, and more continuous high-value structures, rendering the processed data more suitable as input for neural networks. While localized elevated values on the left remain, their overall influence is minimal.

To assess whether data processing introduces spurious correlations that might result in false detection of regulatory relationships, Fig.2 presents time-lag correlation matrix heatmaps for two gene pairs, MCM2–ALG9 and MCM4–MYCN, which are not known to exhibit regulatory interactions, under different data processing methods. After applying the sliding window method, the heatmaps in Figures2(a) and 2(b) continue to support the conclusion that no regulatory relationships exist, though some differences can be observed. For MCM2–ALG9, the gene expression patterns display a modest degree of similarity, whereas for MCM4–MYCN, there is no evidence of positive correlation between the two genes. MCM2–ALG9 exhibits only a few localized high-value regions, while MCM4–MYCN shows almost none, further indicating the absence of meaningful regulatory interactions.

Following mean aggregation, Figure 2(c) reveals discontinuous horizontal high-value regions in the middle of the heatmap for MCM2–ALG9, suggesting that early-to-mid-phase expression of MCM2 could influence the entire expression window of its paired gene. This observation warrants careful interpretation, as it may indicate that mean aggregation introduces weak, extraneous correlations. Nevertheless, the image features remain clearly distinguishable from those of gene pairs with confirmed regulatory relationships. For MCM4–MYCN, Figure 2(d) continues to show no prominent high-value regions; negative correlations are further reduced, with only faint positive correlations appearing in peripheral areas of the heatmap.

After applying weak correlation filtering and correlation enhancement, Figure 2(e) for MCM2–ALG9 displays no concentrated high-value clusters, with correlations scattered diffusely across the heatmap, making it difficult to infer coherent regulatory signals. For MCM4–MYCN, Figure 2(f) exhibits broadly distributed patchy high-value regions, with low-value areas concentrated in the middle-right portion. This may be a byproduct of the data transformation procedure—specifically, squaring and subsequently square-rooting correlation values—which can artificially amplify correlations, converting some negative correlations into positive ones and inflating their magnitudes. However, despite this amplification, the high-value regions remain too diffuse to yield interpretable regulatory insights.

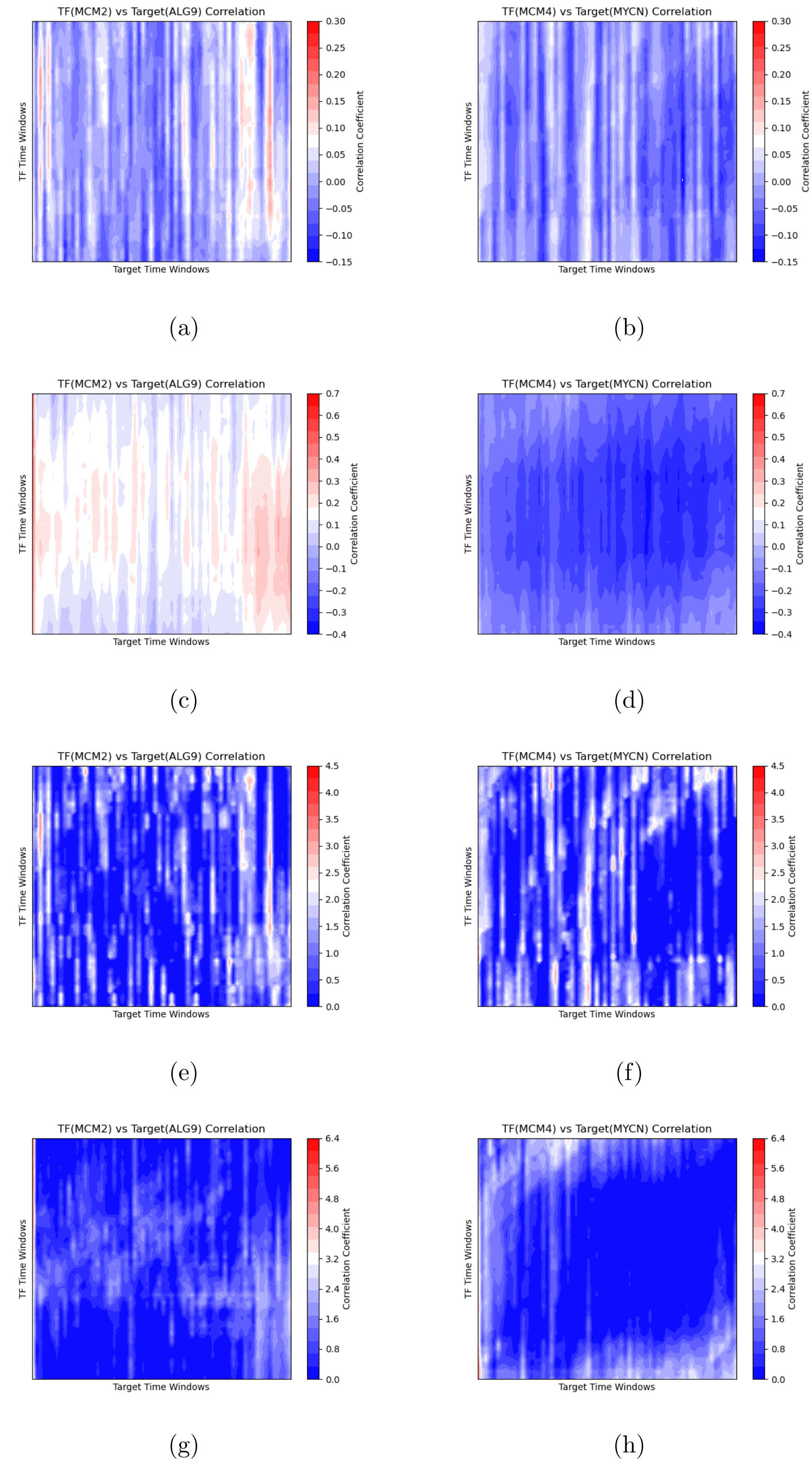

SFigure 2. Time-lagged correlation matrix heatmap for non-regulated gene pairs.

When all processing methods are combined, Figure 2(g) still shows patchy, fragmented high-value regions for MCM2–ALG9. Although weaker correlations have been filtered out, the remaining high-value patterns are discontinuous and lack the coherence seen in gene pairs with true regulatory links. For MCM4–MYCN, the heatmap in Figure 2(h) exhibits almost no central high-value regions, with only a few peripheral hotspots, reaffirming the absence of a discernible regulatory relationship.

While visual inspection alone cannot definitively determine the specific contributions of individual gene segments, comparison of Figures 2(g) and 2(h) with the heatmaps of gene pairs that do possess regulatory interactions reveals clear distinctions. These results suggest that the applied data processing strategies do not artificially generate false regulatory signals and can effectively differentiate between gene pairs with and without true regulatory relationships.

4 Predicted Gene Regulatory Networks

Fig. 4 shows the predicted GRNs, including the GRNs for the mHSC-L (500), mHSC-L (1000), mHSC-GM (500), mHSC-GM (1000) dataset, hESC (500), hESC (1000), hHep (500), and hHep (1000) datasets.

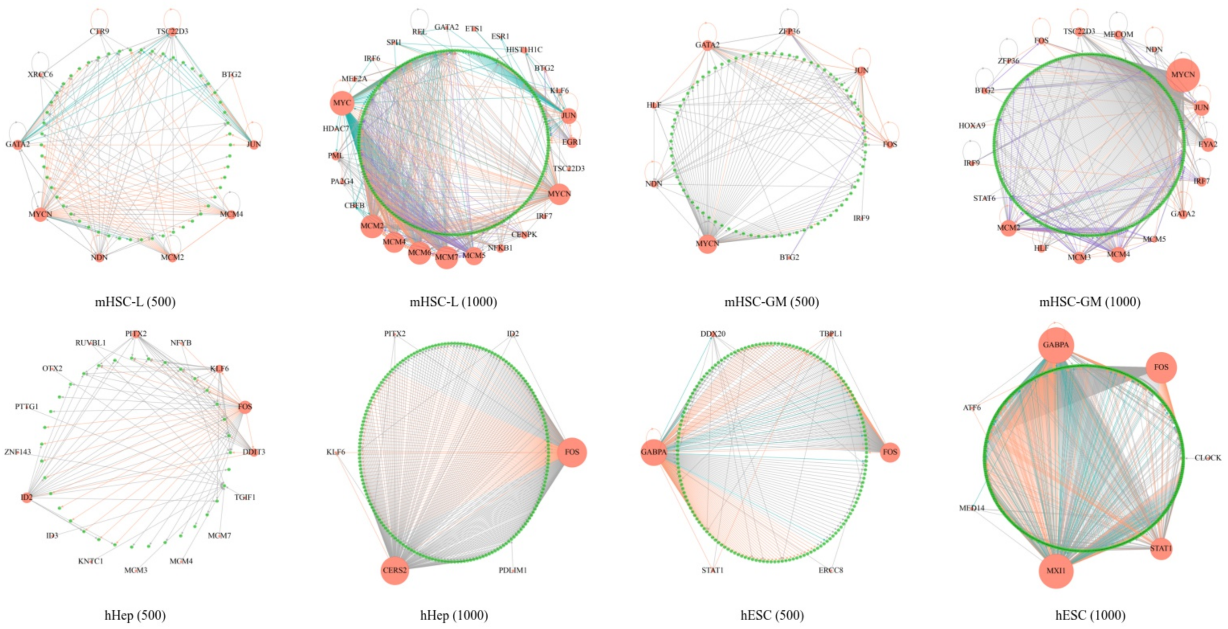

SFigure 3. Diagrams of predicted mouse and human gene regulatory networks using dataset mHSE-E(500).

STable 4. Method performance with different similarity measures using dataset mHSC-E.

Measure	AUROC	AUPR
MI	0.831116	0.389022
Spearman	0.837618	0.345493
Kendall	0.82604	0.39707
Pearson r	0.84046	0.47432

5 Evaluation Metrics: Precision@k, Early Precision, and FDR-Controlled Subnetworks

In addition to global ranking metrics such as AUROC and AUPR, we incorporated several early-retrieval and statistical-confidence metrics that better reflect the practical requirements of gene regulatory network (GRN) inference. These include Precision@k, Early Precision, and false-discovery-rate (FDR) controlled subnetworks. Their definitions and mathematical formulations are provided below.

5.1. Precision@k

Precision@k evaluates the proportion of true regulatory interactions among the top k highest-confidence predictions. Let the predicted edges be sorted in descending order of confidence,

$$\hat{E} = \{e_1, e_2, \ldots, e_N\},$$

and let E^+ denote the set of true regulatory interactions. Considering only the top k predicted edges,

$$\hat{E}_{[1:k]} = \{e_1, e_2, \ldots, e_k\},$$

Precision@k is defined as

$$\text{Precision@}k = \frac{|\hat{E}_{[1:k]} \cap E^+|}{k}.$$

This metric emphasizes the correctness of the highest-ranked predictions, which are typically prioritized for biological validation.

5.2. Early Precision

Early Precision measures the prediction accuracy within the top αN fraction of ranked edges, where $0 < \alpha < 1$. This metric captures early retrieval performance at a fixed proportion of the ranked list. Formally, the top αN predictions are

$$\hat{E}_{[1:\alpha N]},$$

and Early Precision is defined as

$$\text{EarlyPrecision}(\alpha) = \frac{|\hat{E}_{[1:\alpha N]} \cap E^+|}{\alpha N}.$$

Commonly used values include $\alpha = 0.05$ and $\alpha = 0.10$, corresponding to Early Precision@5% and Early Precision@10%. This metric highlights the model's ability to retrieve biologically meaningful interactions at the top of the ranked list.

5.3. FDR-Controlled Subnetworks

To obtain a high-confidence subset of GRN predictions, we applied false-discovery-rate (FDR) control using the Benjamini–Hochberg (BH) procedure. Suppose each predicted edge is assigned a p-value, denoted by

$$p_1, p_2, \ldots, p_N.$$

These p-values are first sorted in ascending order,

$$p_{(1)} \leq p_{(2)} \leq \cdots \leq p_{(N)}.$$

Given a desired FDR threshold $q \in (0, 1)$, the BH step-up procedure identifies the largest index k such that

$$p_{(k)} \leq \frac{k}{N} q.$$

All edges with p-values satisfying $p_{(i)} \leq p_{(k)}$ are declared statistically significant. The resulting FDR-controlled subnetwork is defined as

$$E_{\text{FDR} \leq q} = \{e_i : p_i \leq p_{(k)}\}.$$

This subnetwork provides a set of high-confidence regulatory interactions suitable for downstream analyses such as hub detection, network motif enrichment, or pathway analysis.

STable 5. Performance of DeepCE using different datasets.

	Precision			Early-precision			FDR-controlled Sub-networks		
Dataset	0.01	0.05	0.1	0.01	0.02	0.05	0.05	0.1	0.2
mHSC-E(1000)	0.584	0.376	0.232	0.584	0.523	0.376	1	0.916	0.805
mHSC-GM(1000)	0.512	0.328	0.209	0.512	0.474	0.328	1	1	1
mHSC-L(1000)	0.511	0.232	0.162	0.511	0.352	0.232	1	1	0.8
mHSC-E(500)	0.742	0.508	0.433	0.742	0.647	0.508	1	1	0.8
mHSC-GM(500)	0.611	0.543	0.375	0.611	0.648	0.543	1	1	0.8
mHSC-L(500)	0.443	0.376	0.262	0.445	0.501	0.376	1	1	1

STable 6. Performance of different models based on dataset mHSE-E(500). The blank entries denote that no gene pair met the threshold for statistical significance.

	Precision			Early-precision			FDR-controlled Sub-networks		
Dataset	0.01	0.05	0.1	0.01	0.02	0.05	0.05	0.1	0.2
DeepCE	0.742	0.508	0.433	0.742	0.647	0.508	1	1	0.8
GENELink	1	0.923	0.9615	1	1	0.936	0.966	0.9	0.804
DGRNS	0.583	0.595	0.516	0.583	0.562	0.595			
DeepSEM	0.109	0.0685	0.050	0.109	0.093	0.093			

References

[1] J Gray Camp, Keisuke Sekine, Tobias Gerber, et al. Multilineage communication regulates human liver bud development from pluripotency. *Nature*, 546(7659):533–538, 2017.

[2] Li-Fang Chu, Ning Leng, Jue Zhang, et al. Single-cell rna-seq reveals novel regulators of human embryonic stem cell differentiation to definitive endoderm. *Genome biology*, 17:1–20, 2016.

[3] Carrie A Davis, Benjamin C Hitz, Cricket A Sloan, et al. The encyclopedia of dna elements (encode): data portal update. *Nucleic Acids Research*, 46(D1):D794–D801, 2018.

[4] Luz Garcia-Alonso, Christian H Holland, Mahmoud M Ibrahim, Denes Turei, and Julio Saez-Rodriguez. Benchmark and integration of resources for the estimation of human transcription factor activities. *Genome Research*, 29(8):1363–1375, 2019.

[5] Heonjong Han, Jae-Won Cho, Sangyoung Lee, et al. Trrust v2: an expanded reference database of human and mouse transcriptional regulatory interactions. *Nucleic Acids Research*, 46(D1):D380–D386, 2018.

[6] Zhi-Ping Liu, Canglin Wu, Hongyu Miao, and Hulin Wu. Regnetwork: an integrated database of transcriptional and post-transcriptional regulatory networks in human and mouse. *Database*, 2015:bav095, 2015.

[7] Sonia Nestorowa, Fiona K Hamey, Blanca Pijuan Sala, et al. A single-cell resolution map of mouse hematopoietic stem and progenitor cell differentiation. *Blood, The Journal of the American Society of Hematology*, 128(8):20–31, 2016.

[8] Shinya Oki, Tazro Ohta, Go Shioi, et al. Chip-atlas: a data-mining suite powered by full integration of public chip-seq data. *EMBO Reports*, 19(12):e46255, 2018.

[9] Aditya Pratapa, Amogh P Jalihal, Jeffrey N Law, Aditya Bharadwaj, and TM Murali. Benchmarking algorithms for gene regulatory network inference from single-cell transcriptomic data. *Nature Methods*, 17(2):147–154, 2020.

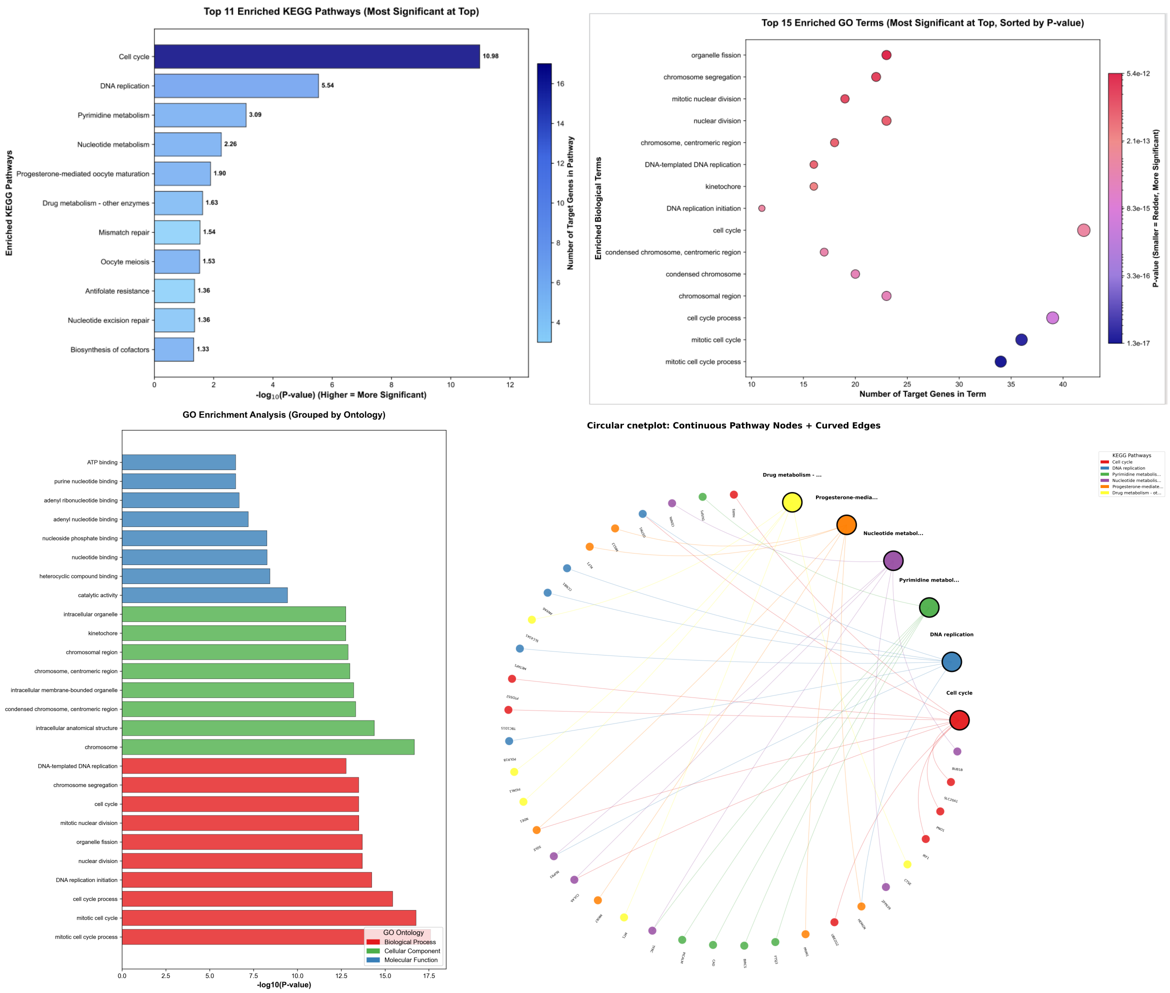

SFigure 4. Functional enrichment analysis of the top identified genetic regulations using mHSE-E(500). (a) Barplot showing the enrichment scores of significantly enriched KEGG pathways. (b) Dotplot visualizing the enrichment analysis results, combining statistical significance with the magnitude of enrichment. (c) Integrated visualization of Gene Ontology enrichment results across biological processes, cellular components, and molecular functions. (d) Cnetplot displaying the relationships between genes and the top enriched pathways, where genes are connected to the biological pathways they belong to.

[10] Alicia T Specht and Jun Li. Leap: constructing gene co-expression networks for single-cell rna-sequencing data using pseudotime ordering. *Bioinformatics*, 33(5):764–766, 2017.

[11] Damian Szklarczyk, Annika L Gable, David Lyon, et al. String v11: protein–protein association networks with increased coverage, supporting functional discovery in genome-wide experimental datasets. *Nucleic Acids Research*, 47(D1):D607–D613, 2019.

[12] Huilei Xu, Caroline Baroukh, Ruth Dannenfelser, et al. Escape: database for integrating high-content published data collected from human and mouse embryonic stem cells. *Database*, 2013:bat045, 2013.

[13] Mengyuan Zhao, Wenying He, Jijun Tang, Quan Zou, and Fei Guo. A hybrid deep learning framework for gene regulatory network inference from single-cell transcriptomic data. *Briefings in bioinformatics*, 23(2):1–13, 2022.
